# Release Kinetics of Papaverine Hydrochloride from Tablets with Different Excipients

**DOI:** 10.3797/scipharm.1310-19

**Published:** 2014-05-16

**Authors:** Regina Kasperek, Andrzej Polski, Łukasz Zimmer, Ewa Poleszak

**Affiliations:** Department of Applied Pharmacy, Faculty of Pharmacy, Medical University of Lublin, 1 Chodzki Str. 20-093, Lublin, Poland.

**Keywords:** Excipients, Kinetic model, Papaverine, Release study, Tablets

## Abstract

The influence of excipients on the disintegration times of tablets and the release of papaverine hydrochloride (PAP) from tablets were studied. Ten different formulations of tablets with PAP were prepared by direct powder compression. Different binders, disintegrants, fillers, and lubricants were used as excipients. The release of PAP was carried out in the paddle apparatus using 0.1 N HCl as a dissolution medium. The results of the disintegration times of tablets showed that six formulations can be classified as fast dissolving tablets (FDT). FDT formulations contained Avicel PH 101, Avicel PH 102, mannitol, (3-lactose, PVP K 10, gelatinized starch (CPharmGel), Prosolv Easy Tab, Prosolv SMCC 90, magnesium stearate, and the addition of disintegrants such as AcDiSol and Kollidon CL. Drug release kinetics were estimated by the zero- and first-order, Higuchi release rate, and Korsmeyer-Peppas models. Two formulations of the tablets containing PVP (K10) (10%), CPharmGel (10% and 25%), and Prosolv Easy Tab (44% and 60%) without the addition of a disintegrant were well-fitted to the kinetics models such as the Higuchi and zero-order, which are suitable for controlled- or sustained-release.

## Introduction

The successful formulation of a stable, effective dosage form and bioavailability of the active substances depend on the selection of the excipients. Usually, in order to prepare tablets, it is necessary to add excipients such as binders (e.g. microcrystalline cellulose, cellulose ethers, starch, polyvinylpyrrolidone, polyethylene glycol), fillers (e.g. lactose, sucrose, glucose, mannitol, sorbitol), disintegrants (crospovidone, sodium starch glycolate, croscarmellose sodium), glidants (fumed silica, colloidal silicone dioxide), and lubricants (magnesium stearate, stearic acid, sodium stearyl fumarate). Most excipients (e.g. microcrystalline cellulose, starch) fulfil multiple functions [1–3]. Microcrystalline cellulose (MCC) is available under the brand names Avicel, Emcocel, Vivacel etc. The types of MCC vary in the size of the particle. Larger particles of microcrystalline cellulose (PH 102, PH 302, and SMCC 90) have better flowability and lubricity, but lower compressibility. Denser particles of MCC show improved flowability, reduced lubricity, and reduced compressibility [4]. Commercially available blends of MCC with colloidal silicon dioxide ensure better flowability and compressibility compared with Emcocel and Avicel PH 101 or a physical mixture of MCC with colloidal silicon dioxide [5]. In order to prepare the tablets by direct compression, modified forms of cellulose PH 102 manufactured and their crosslinked form with the addition of stearic acid as a lubricant can be used [6].

Lactose is commonly used as a filler in tablets. Lactose from different suppliers exhibits different properties; therefore, it could not be used interchangeably in direct compression formulations. Lactose may occur in different forms such as α-lactose monohydrate, anhydrous α-lactose, anhydrous β-lactose, spray-dried lactose, or agglomerated lactose. Lactose is available in different mixtures e.g. lactose monohydrate and maize starch [3], α-lactose monohydrate and cellulose [7], α-lactose monohydrate, polyvinyl pyrrolidone (Kollidon 30), and crospovidone (Kollidon CL) [8].

To improve or change the properties of starch, gelatinized starch was prepared by modifying the chemical. Gelatinized starch has a high rate of flow and allows us to obtain a solid oral dosage form using direct compression. It is also used as a binding agent in wet granulation [9]. It should be emphasized that gelatinized corn starch has a high hygroscopicity [10]. Alebiowu and Itiola [11] reported that tablets containing gelatinized starch, microcrystalline cellulose, lactose, and calcium hydrogen phosphate have high hardness. Ready-mix adjuvants are helpful in the process of pre-formulation and facilitate the production of tablets with desired properties.

In the pharmaceutical industry, dissolution testing is a very important tool in drug product development and is a means of a quality control procedure. In product development, it supports formulation selection, enables the analysis of the combined effects such as the drug, excipient, or process properties in order to evaluate the effect of these changes on biopharmaceutical characteristics, and is used in comparative studies of formulations [12].

Several mathematical models have been proposed to describe the release profiles of drugs from various systems [13, 14]. Methods based on the analysis of variance, model-independent methods, and model-dependent methods were proposed to compare dissolution profiles. The analysis of variance presents the differences between the averages of the two drug release data sets. Fit factors (difference and similarity) are the prime examples of model-independent methods. Model-dependent methods allowed us to apply mathematical equations that help observe the physical and chemical phenomena which influenced the drug release [12, 15]. The dissolution process is related to the physical and chemical characteristics of the drug, drug product formula, dosage form, and to the parameters of dissolution testing. The development of controlled or sustained release delivery systems is a tool for optimizing the therapeutic effect by maximizing the bioavailability of conventional drugs and reducing side effects [12, 16]. The release of the drug from a sustained release formulation is controlled by various factors through different mechanisms such as diffusion, erosion, or osmosis [17]. A water-soluble drug incorporated into a hydrophilic matrix is released mainly by a diffusion-controlled process, whereas for a poorly water-soluble compound, the principal mechanism of release is a function of erosion of the matrix that carries the drug [15]. The factors involved in the release of a soluble drug from an insoluble matrix tablet were extensively investigated. These factors are drug solubility and concentration in the tablet, the drug diffusivity, and the tablet porosity and tortuosity [18]. The Higuchi model describes drug release through the diffusion mechanism and it is used to describe drug dissolution from systems such as matrix tablets containing water-soluble drugs. The Hixson-Crowell model assumes that drug release is limited by the dissolution rate of the particles rather than by diffusion through the polymer matrix [15]. The Korsmeyer-Peppas model can be used to characterize the drug release mechanisms as Fickian diffusion [19].

Papaverine hydrochloride was selected as the model drug. It is well-soluble in acidic medium and its solubility increases proportionally with the decrease in pH of the medium [20, 21]. Papaverine hydrochloride is a spasmolytic drug which can be found in opium as its main isoquinoline alkaloid. It is used in bile, intestinal, and renal spikes [22, 23].

In this study, different formulations of tablets were employed and dissolution tests of papaverine hydrochloride were carried out to investigate the kinetics of the active substance release.

## Experimental

### Materials and Reagents

Papaverine hydrochloride (PAP) was the product of Farm-Impex Sp. J., Poland. Polyvinylpyrrolidone K 10 (PVP 10), microcrystalline cellulose (Avicel PH 101, Avicel PH 102), α-lactose (lactose), crospovidone CL (Kollidon CL), magnesium stearate (MS), and mannitol were produced by Sigma-Aldrich Chemmie Gmbh, Germany. Croscarmellose sodium (AcDiSol) was the product of BioPolymer, Belgium and gelatinized starch (CPharmGel) was the product of CargillBenelux BV, Netherlands. The manufactured mixture called Prosolv EasyTab SP (Prosolv) containing microcrystalline cellulose, colloidal silicon dioxide, sodium starch glycolate, and sodium stearyl fumarate was produced by JRS Pharma Gmbh & CO KG, Germany. The manufactured mixture called Prosolv SMCC 90 containing microcrystalline cellulose and colloidal silicon dioxide (CMSi), and manufactured by JRS Pharma Gmbh & CO KG, Germany, was also used. All other reagents and solvents were of analytical grade, distilled water was freshly distilled.

## Methods

### Preparation of Tablets

Tablets (T1-T10) were prepared by direct compression according to the formulas given in Table 1. Each tablet contained 40 mg of papaverine hydrochloride and weighed 200 mg.

**Tab. 1.** Composition of tablet formulations containing PAP

Component	Quantity in percent per tablet at 200 mg of weight									
	T1	T2	T3	T4	T5	T6	T7	T8	T9	T10
PAP	20.0	20.0	20.0	20.0	20.0	20.0	20.0	20.0	20.0	20.0
PVP 10	7.5	7.5	–	–	5.0	7.5	10.0	10.0	7.5	10.0
Avicel PH 101	30.0	–	–	–	–	–	–	–	–	–
Avicel PH 102	–	30.0	–	–	20.0	–	–	–	–	–
Lactose	36.5	36.5	–	–	29.0	56.5	–	22.0	44.5	–
Mannitol	–	–	–	20.0	20.0	–	–	–	–	–
AcDiSol	5.0	5.0	–	5.0	–	5.0	–	–	2.0	–
CPharmGel	–	–	–	–	–	10.0	10.0	10.0	25.0	25.0
Kollidon CL	–	–	–	–	5.0	–	–	2.0	–	–
Prosolv	–	–	80.0	–	–	–	60.0	–	–	44.0
CMSi	–	–	–	54.0	–	–	–	35.0		
MS	1.0	1.0	–	1.0	1.0	1.0	–	1.0	1.0	1.0

The powders of the components were sieved through a 0.710 mm mesh screen and mixed manually. The obtained powder mixtures were compressed using a press tablet machine (Erweka, Germany) with a 9 mm punch at a compression force of around 3 kN.

### Physical Studies

#### Weight Uniformity Test

From each batch, 20 tablets were randomly selected and weighted together by using a weighing balance (Mettler AT 201, Switzerland). Their mean weight was calculated and then they were individually weighed.

#### Tablet Thickness

Tablet thickness was measured using a Vernier Caliper (Digital Caliper 0-150 mm, Comparator).

#### Friability Test

A friabilator (Erweka TAR 120, Germany) was used in the test. Twenty tablets from each series were weighed and placed into the friabilator. The machine was set to 25 rpm for 4 min. After that, they were reweighed. The friability of the tablets was calculated according to Equation 1:


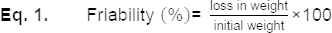


#### Hardness Test

The hardness tester (AEG Type AP 56 N2, Germany) was used to study the hardness of the tablets. Six tablets were randomly selected from each series. The breaking force needed to crush the tablet was observed.

#### Tablet Disintegration Time Assay

Disintegration times were determined by using the European Pharmacopeia (Ph Eur.) Apparatus [1] (Erweka Type ZT 222, Germany). Six tablets were randomly selected from each batch and were put into a basket-rack in a vessel with water at 37°C which then was covered with a disk. After the apparatus was turned on, the disintegration time of the tablets was observed. For non-modified tablets, the disintegration time should be no longer than 15 min, for the fast-dissolving tablets no longer than 3 min [1].

#### Drug Content Analysis

The PAP quantification assay was carried out using a UV spectrophotometric method adapted from the pharmacopoeial method [2].

Ten tablets from each batch were randomly selected and crushed together. The weighed amount of 200 mg powder was transferred into a 100 ml volumetric flask and 50 ml 0.1 N HCl was added. The flask was shaken for five minutes and diluted with 0.1 N HCl. Next, the mixture was filtered through a Whatman filter (0.45 um pore size) and 2 ml of the solution was transferred into a 100 ml volumetric flask and diluted with 0.1 N HCl. The absorbance of this solution was determined by UV spectrophotometry at 251 nm (Omega UV - VIS, Thermo Scientific, England). The PAP concentration was calculated according to the following equation: y = 0.146 x + 0.106 (r^2^ = 0.999), obtained from a standard curve of PAP (n = 5). This method obeys Beer’s Law within the concentration range of 2.5–20 μg/ml for PAP. The experiment was repeated six times (n = 6).

### Release Studies

The dissolution test was carried out in a Ph Eur. Apparatus 2 [1] (Erweka, Germany) called a paddle apparatus. For the test, 900 ml of 0.1 N HCI maintained at 37 ± 0.5°C was used as a dissolution medium according to the pharmacopeial requirements for non-coated tablets. Each tablet was placed in each of the six vessels of the paddle apparatus and rotated at 75 rpm [1, 2]. After appropriate intervals of time, 2 ml samples were collected and an equivalent amount of 0.1 N HCl (2 ml) was added to the dissolution medium. Each solution containing the drawn samples was mixed and analyzed spectrophotometrically at 251 nm. The amount of the released substance was calculated by reference to a Beer’s plot based on the calibration curve.

Statistical analysis was carried out using SAS 9.1.3 (SAS Institute, Cary, NC, USA). The data obtained were subject to statistical analysis using one-way ANOVA and a *p* value of <0.05 was considered as statistically significant.

### Drug Release Kinetics

The release kinetics of the drug were evaluated by plotting in various kinetic models: zero-order (Eq. 2) as a cumulative percentage of drug release vs. time, first-order (Eq. 3) as a log of the amount of the drug remaining to be released vs. time, and Higuchi’s model (Eq. 4) as a cumulative percentage of the drug release vs. the square root of time.

The zero-order kinetics describes the systems where the drug release is independent of its concentration.


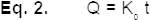


where *Q* is the amount of the drug released in time t, *K_0_* is the zero-order rate constant expressed in units of concentration [24].

The first-order kinetics describes the release, where the release rate is concentration-dependent.


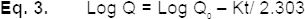


where *Q* is the amount of the drug released in time t, *Q*_0_ is the initial concentration of the drug and *K* is the first-order rate constant [25].

Higuchi’s model describes the release of drugs from the insoluble matrix as a square root of the time-dependent process based on Fickian diffusion.


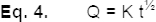


where *Q* is the amount of the drug released in time t, *K* is the constant reflecting the design variables of the system [13].

To evaluate the mechanism of drug release from the tablets, the data of drug release were plotted in Korsmeyer et al.’s equation (Eq. 5) as a log of the cumulative percentage of the drug released vs. log time, and the exponent n value was calculated from the slope of the straight line.


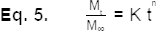


For the cylindrical matrix tablets, if the exponent n = 0.45, then the drug release mechanism is Fickian diffusion, and if 0.45 < n> 0.89, then it is non-Fickian diffusion. An exponent value of 0.89 is indicative of case II transport or typical zero-order release, n > 0.89 is super case-II transport [19].

Statistical and kinetic analyses were made using Statistica 8.0 software.

## Results and Discussion

As shown in Table 2, all prepared tablets fulfilled pharmacopoeical requirements such as the average weight, hardness, and assay of the drug content [1, 2].

In our study, six formulations with disintegration times shorter than 3 min (T1-T6) were obtained, so these tablets can be classified as fast disintegrating tablets (FDT) [1]. The disintegration times for tablets T8 and T9 did not exceed 15 min, as recommended by Ph Eur. [1]. Only the two formulations T7 and T10 had excessively long disintegration times which amounted to 34.4 min and 25.43 min, respectively.

**Tab. 2.** Characteristics of PAP tablets

Formula	Weight average (mg)^a^	Size (mm)^a^	Disintegration time (min:sec)^b^	Breaking force (N)^b^	Friability (%)^a^	Drug content (%)^c^
T1	208 ± 4.0	3.16 ± 5.3	1:03	43 ± 6.38	0.8534	99.08 ± 2.25
T2	208 ± 4.0	3.00 ± 0.0	1:58	43 ± 6.60	0.1791	98.08 ± 3.56
T3	209 ± 4.5	3.20 ± 6.7	0:17	36 ± 7.59	0.3004	100.61 ± 1.23
T4	207 ± 3.5	3.12 ± 4.0	0:12	41 ± 3.58	0.7279	91.49 ± 2.31
T5	204 ± 2.0	2.94 ± 2.0	0:27	40 ± 1.52	0.8920	102.61 ± 5.45
T6	203 ± 1.5	2.78 ± 5.3	2:10	43 ± 9.71	0.2180	90.34 ± 4.34
T7	203 ± 1.5	2.94 ± 2.0	34:40	50 ± 2.58	0.0483	99.02 ± 1.99
T8	204 ± 2.0	3.00 ± 0.0	11:42	43 ± 1.97	0.3227	101.61 ± 2.45
T9	201 ± 0.5	3.12 ± 4.0	7:52	56 ± 2.23	0.2489	100.22 ± 5.67
T10	207 ± 3.5	3.16 ± 5.3	25:43	46 ± 1.65	0.2455	98.72 ± 3.01

^a^ The values represent the mean of twenty determinations;

^b^ The values represent the mean of six determinations;

^c^ The values represent the mean of ten determinations ± standard deviation.

These results showed that the addition of excipients had a significant effect on the disintegration time of the tablets. In general, a sustained release of PAP was observed in formulations T7 and T10 containing 10% of PVP 10 and CPharmGel (10% and 25%, respectively) and Prosolv Easy Tab (44% and 60%, respectively). Having analyzed the composition of these tablets, it may be assumed that the lack of a disintegrant is responsible for the longer disintegration times of the tablets. Although Prosolv contains the addition of sodium starch glycolate, which is a disintegrant, in the presence of other excipients, the disintegration times of the tablets were longer. Formulation T3 contained, besides the active substance, the addition of Prosolv as the only excipient and the disintegration time took only 17 seconds. It can be assumed, then, that the longer disintegration of tablets affected the additives of the binders such as CPharmGel and PVP 10 in T7 and T10.

Although formulations T6, T8, and T9 also contained CPharmGel in the amount of 10–25%, their disintegration times were faster. In these formulations, there were also different amounts (7.5–10%) of PVP 10 and 22–56.5% of lactose. It should be noted that these formulations contained the addition of disintegrants. In T6 and T9, 2–5% of AcDiSol was used and their disintegration times were 2.1 min and 7.52 min, respectively. Formulation T8 contained 2% of Kollidon CL that probably influenced the disintegration time which amounted to 11.42 min. Sallam et al. [26] tested the effects of four fast disintegrants on the dissolution of terfenadine from tablets. The relative efficiency of improvement was in the decreasing order: crospovidone, AcDiSol, sodium starch glycolate, low substituted hydroxypropylcellulose [26]. The use of blends with a disintegrant facilitated the production of tablets disintegrating rapidly [27].

The relationship between the disintegration time of the tablets and the amount or type of disintegrants was investigated by Zhao and Augsburger [28]. Disintegration testing without a disc revealed a significant increase in the disintegration time for tablets formulated with dry granulated sodium starch glycolate and crospovidone, but not for AcDiSol and all wet granulated disintegrants.

Increasing amounts of lactose enhanced the tablet hardness and disintegration time, therefore an appropriate amount of a disintegrant should be added to the composition of tablets [29–31]. In our study, in tablets T1, T2, T5 lactose in the amount of about 30% was added but disintegration times of the tablets were no longer than 3 min. The addition of 22%, 44.5%, and 56.6% of lactose to T8, T9, T6, respectively, caused a prolongation of the disintegration times only for T8 and T9. All of the analyzed formulations contained a disintegrant. It showed that the prolongation of the disintegration time corresponds to the addition of other excipients, particularly CPharmGel in T6. Late et al. [32] demonstrated that lubricants such as magnesium stearate and glyceryl behenate had a significant effect on the disintegration time and hardness of granisetron hydrochloride tablets containing β-cyclodextrine, lactose, and mannitol, but talc and stearic acid were not of a significant influence.

Only the two formulations T6 and T9 did not contain microcrystalline cellulose and their disintegration times were 3 min and 15 min, respectively. It showed that the addition of other excipients besides microcrystalline cellulose affected the disintegration times. Using Avicel PH 101 or Avicel PH 102 in T1 and T2 did not affect the physical properties of the tablets, disintegration times, and the release of PAP. Lahdenpaa et al. [33] demonstrated that tablets containing a higher percentage of Avicel PH101 exhibited higher crushing strength and lower disintegration time, whereas tablets containing Avicel PH102 and PH200 showed lower crushing strength, shorter disintegration time, and small weight variation.

**Fig. 1.**
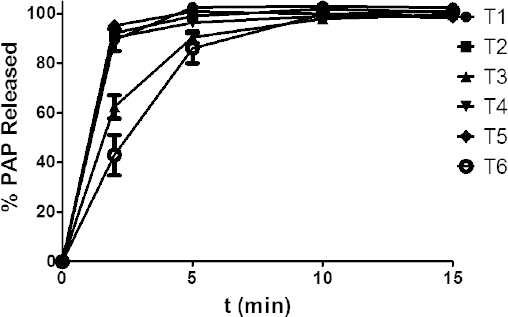
Mean dissolution profiles of PAP from tables T1–T6 (mean values n=6, ± SD, *p*>0.05)

**Fig. 2.**
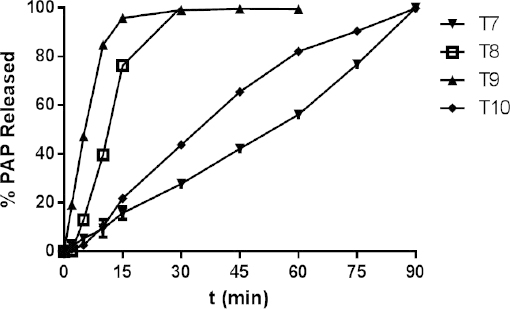
Mean dissolution profiles of PAP from tables T7–T10 (mean values n=6, ± SD*, p*>0.05)

Similarly, the release profiles of PAP presented in figs. 1 and 2 show that from the six formulations T1–T6, over 80% of PAP was released within 5 min and from T8 and T9 within 20 min and 10 min, respectively, which corresponded to the pharmacopoeial recommendation for non-modified tablets (release within 45 min) [1, 2].

Within 45 min, 80% of PAP was not released from tablets T7 and T10 which showed that the formulations displayed a prolonged release of PAP.

Testing conditions such as the type of the apparatus, agitation speed and volume, composition, and temperature of the dissolution medium influenced the drug release from the tablets [34]. In our study, the testing conditions were constant and identical for all formulations which shows that the release process of the active substance was dependent on the compositions of the tablets. Swellable matrix tablets are activated by water and the drug release is controlled by the interaction between water, the polymer, and the drug. Hydratation of the polymer results in the formation of a gel layer that controls the drug release rate [35]. In our study, the addition of CPharmGel caused a prolonged release of the drug especially from two formulations: T7 and T10. However, a prolonged process of the release depended on other ingredients of the formulations because the gel layer responsible for the prolonged release may not occur at low concentrations [36]. *In vitro* drug dissolution from the matrix tablets is significantly influenced by the amount of polymer in the formulation [12].

The obtained drug release data were analyzed by zero-order, first-order, and Higuchi and Korsmeyer–Peppas models to discover the mechanism of drug release from the formulations. The release rate constants were calculated from the slope of the appropriate plot, and the determination coefficient (r^2^) was determined (Table 3).

**Tab. 3.** Kinetic parameters of PAP release from tablets

Tablets	Zero-order		First-order		Higuchi		Korsmeyer–Peppas	
	K_0_	r^2^	K^1^	r^2^	K_H_	r^2^	*n*	r^2^
T1	0.127	0.157	0.028	0.498	1.407	0.277	0.033	0.475
T2	0.051	0.064	0.020	0.122	0.681	0.162	0.018	0.356
T3	0.492	0.314	0.054	0.294	5.025	0.474	0.130	0.667
T4	0.077	0.150	0.031	0.362	0.889	0.290	0.021	0.508
T5	0.071	0.294	0.002	0.011	0.456	0.175	0.007	0.116
T6	0.777	0.317	0.082	0.351	7.941	0.478	0.238	0.656
T7	1.059	0.989	0.060	0.551	11.382	0.928	0.953	0.999
T8	3.614	0.906	0.184	0.954	26.452	0.961	3.252	0.946
T9	2.555	0.655	0.163	0.949	20.082	0.814	0.928	0.998
T10	1.198	0.972	0.055	0.780	13.403	0.987	1.486	0.983

In this study, the *in vitro* release profiles from formulations T7, T8, T9, and T10 containing CPharmGel can be described by mathematical models to evaluate the release kinetics of PAP.

It was found that the PAP release from tablets T7 and T10 was best explained by the Higuchi model (r^2^ = 0.928 and 0.987, respectively), and zero-order model (mean r^2^ = 0.989 and 0.972, respectively). This indicates that the release of the drug from the matrix is a square root of the time-dependent process characteristic for the release of the soluble form of the drug containing hydrophilic polymers, and the diffusion of the drug is relatively slow. The zero-order release kinetics indicate that the concentration was nearly independent of the drug release profile. The release profile of T8 showed the highest linearity with the Higuchi model (r^2^= 0.961), followed by first-order kinetics (r^2^= 0.954). This indicates that the release of the drug from the matrix is a square root of the time-dependent process describing the drug release rate relationship with the concentration of the drug. The formulation T9 can be best explained by a first-order model as the plots showed the highest linearity (r^2^= 0.949).

To find out the mechanism of the drug release, the first 60% of the drug release data was fitted in the Korsmeyer-Peppas model.

The release profiles from formulations T7, T8, T9, and T10 exhibited good linearity with the determination coefficient (r^2^): 0.999, 0.9946, 0.998, and 0.983, respectively. The values of the exponent (n) providing the type of release mechanism were in the range of 0.928 to 3.252, which indicate that a super case II transport refers to the erosion of the polymer and the drug diffusion process. Case II release is the drug transport mechanism associated with stresses and state-transition in hydrophilic glassy polymers which swell in water or biological fluids [19, 37].

Excipients added to ten different formulations significantly influenced the release process of PAP from the tablets. Six of the prepared batches of tablets had disintegration times shorter than 3 min and may be classified as fast dissolving tablets. The four formulations containing the addition of CPharmGel had longer disintegration times in the range from 7.52 min to 34.4 min. The release of PAP showed that eight formulations may be classified as non-modified release and two as prolonged-release ones. The formulations (T7 and T10) containing CPharmGel in the range of 10% to 25%, Prosolv Easy Tab (44-60%), and 10% PVP 10 can be considered as tablets with a prolonged release, because the *in vitro* release profiles of PAP from these formulations were fitted to the kinetic models such as Higuchi’s model and zero-order drug release.

In conclusion, tablets containing polyvinylpyrrolidone (PVP K 10), gelatinized starch (CPharmGel), microcrystalline cellulose, colloidal silicon dioxide, sodium starch glycolate, and sodium stearyl fumarate (Prosolv Easy Tab) may be considered as a controlled- or sustained-release solid dosage form.

## References

[1] European Pharmacopeia 5.0. Council of Europe, Strasbourg. 2005: 225-226, 228-229, 234-235, 627, 2183-2184.

[2] The Polish Pharmacopeia IX. The Polish Pharmaceutical Society, Warsaw. 2011: 355, 359, 836-837, 1633, 1836-1837, 1888-1890, 2616-2619, 2710-2712, 2721-2723, 2726, 2891-2892, 3102-3105, 3240-3241, 3246.

[3] Gohel MC A review of co-processed directly compressible excipients. J Pharm Pharm Sci. 2005; 8: 76–93. http://www.ncbi.nlm.nih.gov/pubmed/15946601 15946601

[4] Hwang R Peck GR A systematic evaluation of the compression and tablets characteristics of various types of microcrystalline cellulose. Pharm Technol. 2001; 24: 112–132.

[5] Luukkonen P Schaefer T Hellen L Juppo A Yliruusi J Rheological characterization of microcrystalline cellulose and silicified microcrystalline cellulose wet masses using a mixer torque rheometer. Int J Pharm. 1999; 188: 181–192. http://dx.doi.org/10.1016/S0378-5173(99)00219-7 10518674 10.1016/s0378-5173(99)00219-7

[6] De la Luz Reus Medina M Kumar V Evaluation of cellulose II powders as a potential multifunctional excipient in tablet formulations. Int J Pharm. 2006; 322: 31–35. http://dx.doi.org/10.1016/j.ijpharm.2006.05.033 16828996 10.1016/j.ijpharm.2006.05.033

[7] Garr JS Rubinstein MH Compaction properties of a cellulose-lactose direct compression excipient. Pharm Tech Int. 1991; 3: 24–27.

[8] Whiteman M Yarwood RJ Evaluation of six lactose-based materials as direct compression tablet excipients. Drug Dev Ind Pharm. 1988; 14: 1023–1040. http://dx.doi.org/03639048809151918

[9] Mimura K Kanada K Uchida S Yamada M Namiki N Formulation study for orally disintegrating tablet using partly pregelatinized starch binder. Chem Pharm Bull. 2011; 59: 959–964. http://dx.doi.org/10.1248/cpb.59.959 21804239 10.1248/cpb.59.959

[10] Callahan JC Cleary GW Elefant M Equilibrium moisture content of pharmaceutical excipients. Drug Dev Ind Pharm. 1982; 8: 355–369. http://dx.doi.org/10.3109/03639048209022105

[11] Alebiowu G Itiola OA Compression characteristics of native and pregelatinized forms of sorghum, plantain, and corn starches, and the mechanical properties of their tablets. Drug Dev Ind Pharm. 2002; 28: 663–672. http://dx.doi.org/10.1081/DDC-120003857 12149958 10.1081/ddc-120003857

[12] Mourao SC da Silva C Bresolin TM Serra CH Porta V Dissolution parameters for sodium diclofenac-containing hypromellose matrix tablet. Int J Pharm. 2010; 386: 201–207. http://dx.doi.org/10.1016/j.ijpharm.2009.11.022 19941944 10.1016/j.ijpharm.2009.11.022

[13] Higuchi T Mechanism of sustained-action medication. Theoretical analysis of rate of release of solid drugs dispersed in solid matrices. J Pharm Sci 1963; 52: 1145–1149. http://dx.doi.org/10.1002/jps.2600521210 14088963 10.1002/jps.2600521210

[14] Bamba M Puisieux F Marty JP Carstensen JT Physical model for release of drug form gelforming sustained release preparations. Int J Pharm: 1979; 3: 87–92. http://dx.doi.org/10.1016/0378-5173(79)90069-3

[15] Costa P Lobo JM Modelling and comparison of dissolution profiles. Eur J Pharm Sci. 2001; 13: 123–133. http://dx.doi.org/10.1016/S0928-0987(01)00095-1 11297896 10.1016/s0928-0987(01)00095-1

[16] Lachman L Lieberman HA Kanig J Teoria e práctica na indústria farmacêutica vol. 2. Lisboa: Fundação Calouste Gulbenkan. 2001: 509–597.

[17] Su SF Chou CH Kung CF Huang J In vitro and in vivo comparison of two diclofenac sodium sustained release oral formulations. Int J Pharm. 2003; 260: 39–46. http://dx.doi.org/10.1016/S0378-5173(03)00237-0 12818808 10.1016/s0378-5173(03)00237-0

[18] Attama AA Adikwu MU Nnamani PO Delivery of diclofenac sodium via non-disintegrating bioadhesive tablets of paraffin wax. S T P Pharm Sci. 2003; 13: 147–150.

[19] Siepmann J Peppas NA Modeling of drug release from delivery system based on hydroxypropyl methylcellulose (HPMC). Adv Drug Deliver Rev. 2001; 48: 139–157. http://dx.doi.org/10.1016/S0169-409X(01)00112-0 10.1016/s0169-409x(01)00112-0 11369079

[20] Miyajima M Koshika A Okada J Kusai A Ikeda M Factors influencing the diffusion-controlled release of papaverine from poly (L-lactic acid) matrix. J Control Release. 1998; 56: 85–94. http://dx.doi.org/10.1016/S0168-3659(98)00076-5 9801432 10.1016/s0168-3659(98)00076-5

[21] Serajuddin AT Rosoff M pH-solubility profile of papaverine hydrochloride and its relationship to the dissolution rate of sustained-release pellets. J Pharm Sci. 1984; 73: 1203–1208. http://dx.doi.org/10.1002/jps.2600730905 6491936 10.1002/jps.2600730905

[22] Kaneda T Hayasaka R Nagai Y Tajima T Urakawa N Nakajyo S Shimizu K Effect of papaverine on twiches in mouse diaphragm. Pharmacology. 2010; 86: 273–280. http://dx.doi.org/10.1159/000320769 20980780 10.1159/000320769

[23] Martindale: The complete drug reference. The Pharmaceutical Press, London 2011: 44.

[24] Hadjiioannou TP Christian GD Koupparis MA Macheras PE Quantitative calculations in pharmaceutical practice and research. VCH Publishers Inc, New York 2002: 345–348.

[25] Bourne DW Pharmacokinetics. In: Banker GS Rhodes CT, eds. Modern Pharmaceutics. 4th ed.; Marcel Dekker Inc, New York 2002: 67–92.

[26] Sallam E Ibrahim H Dahab RA Shubair M Khalil E Evaluation of fast disintegrants in terfenadine tablets containing a gas-evolving disintegrant. Drug Dev Ind Pharm. 1998; 24: 501–507. http://dx.doi.org/10.3109/03639049809085650 9876615 10.3109/03639049809085650

[27] Badgujar BP Mundada AS The technologies used for developing orally disintegrating tablets: a review. Acta Pharm. 2011; 61: 117–139. http://dx.doi.org/10.2478/v10007-011-0020-8 10.2478/v10007-011-0020-8 21684842

[28] Zhao N Augsburger LL The influence of granulation on super disintegrant performance. Pharm Dev Technol. 2006; 11: 47–53. http://dx.doi.org/10.1080/10837450500463828 16544908 10.1080/10837450500463828

[29] Kuno Y Kojima M Nakagami H Yonemochi E Terada K Effect of the type of lubricant on the characteristic of orally disintegrating tablets manufactured using the phase transition of sugar alcohol. Eur J Biopharm. 2008; 69: 986–992. http://dx.doi.org/10.1016/j.ejpb.2008.02.016 10.1016/j.ejpb.2008.02.016 18396020

[30] Kranz H Guthmann C Wagner T Lipp R Reinhard J Development of a single unit extended release formulation for ZK 811 752, a weakly basic drug. Eur J Pharm Sci. 2008; 26: 47–53. http://dx.doi.org/10.1016/j.ejps.2005.04.018 15953712 10.1016/j.ejps.2005.04.018

[31] Hie I Kasa Jr P Dreu R Pintye-Hodi K Srcic S The compressibility and compatibility of different types of lactose. Drug Dev Ind Pharm. 2009; 35: 1271–1280. http://dx.doi.org/10.1080/03639040902932945 19466896 10.1080/03639040902932945

[32] Late SG Yu YY Banga AK Effects of disintegration-promoting agent, lubricants and moisture treatment on optimized fast disintegrating tablets. Int J Pharm. 2009; 365: 4–11. http://dx.doi.org/10.1016/j.ijpharm.2008.08.010 18778759 10.1016/j.ijpharm.2008.08.010

[33] Lahdenpaa E Niskanen M Yliruusi J Crushing strength, disintegration time and weight variation of tablets compressed from three Avicel PH grades and their mixtures. Eur J Pharm Biopharm. 1997; 43: 315–322. http://dx.doi.org/10.1016/S0939-6411(97)00053-2

[34] Abdou H Dissolution. Bioavailability & Bioequivalence. Mack Printing Company, Easton, Pensylvania 1989: 145–172.

[35] Colombo P Bettini R Santi P Peppas NA Swellable matrices for controlled drug delivery: gel-layer behaviour mechanisms and optimal performance. Pharm Sci Technol To. 2000; 3: 198–204. http://dx.doi.org/10.1016/S1461-5347(00)00269-8 10.1016/s1461-5347(00)00269-8 10840390

[36] Li CL Martini LG Ford JL Roberts M The use of hypromellose in oral drug delivery. J Pharm Pharmacol. 2005; 57: 533–546. http://dx.doi.org/10.1211/0022357055957 15901342 10.1211/0022357055957

[37] Ritger PL Peppas NA A simple equation for description of solute release II. Fickian and anomalous release from swellable devices. J Control Release. 1987; 5: 37–42. http://dx.doi.org/10.1016/0168-3659(87)90035-6

